# Research on Voltage Waveform Fault Detection of Miniature Vibration Motor Based on Improved WP-LSTM

**DOI:** 10.3390/mi11080753

**Published:** 2020-07-31

**Authors:** Ruirui Wang, Zhan Feng, Sisi Huang, Xia Fang, Jie Wang

**Affiliations:** School of Mechanical Engineering, Sichuan University, Chengdu 610041, China; w_ruirui@126.com (R.W.); fengzhan2019@gmail.com (Z.F.); 2017223025092@stu.scu.edu.cn (X.F.); wangjie@scu.edu.cn (J.W.)

**Keywords:** fault detection, miniature vibration motor, wavelet packet, LSTM neural network

## Abstract

To solve the problem of vibration motor fault detection accuracy and inefficiency in smartphone components, this paper proposes a fault diagnosis method based on the wavelet packet and improves long and short-term memory network. First, the voltage signal of the vibration motor is decomposed by a wavelet packet to reconstruct the signal. Secondly, the reconstructed signal is input into the improved three-layer LSTM network as a feature vector. The memory characteristics of the LSTM network are used to fully learn the time-series fault feature information in the unsteady state signal, and then, the model is used to diagnose the motor fault. Finally, the feasibility of the proposed method is verified through experiments and can be applied to engineering practice. Compared with the existing motor fault diagnosis method, the improved WP-LSTM diagnosis method has a better diagnosis effect and improves fault diagnosis.

## 1. Introduction

**Figure 1:**
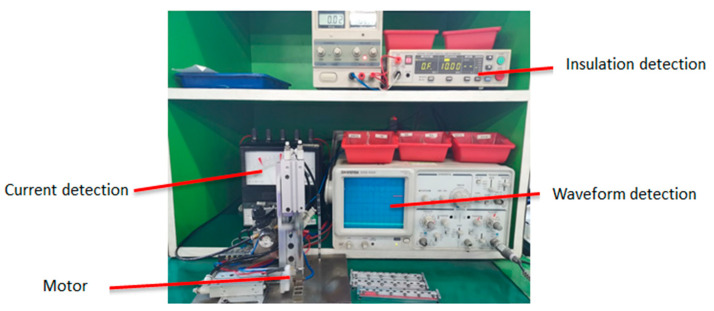
A motor waveform detection station.

Vibration motors are mainly used in mobile phones, tablets, and smart wearable devices. Their main function is to make the device vibrate and give users timely message reminders. With the arrival of the 5G era, mobile phones and tablets have become an indispensable tool for daily office and social affairs, and each mobile phone and tablet has at least one miniature vibration motor. Therefore, the demand for miniature vibration motors is increasing. At present, around 2 billion vibration motors are produced every year in the world, and the total number of motors produced by Chinese domestic manufacturers accounts for more than 80% of them [1]. Domestic manufacturers inspect the motors by observing the waveform of the oscilloscope with the naked eye to judge the quality of the motor. As shown in Figure 1, manual inspection is slow, and the quality of the inspection cannot be guaranteed [2,3]. 

At present, the fault diagnosis methods for motors are mainly divided into traditional detection methods and intelligent diagnosis methods. Traditional detection methods establish fault models by extracting fault features and then, classify the faults. They include wavelet packet decomposition [4,5], comprehensive empirical model State decomposition [6,7], Hilbert transform [8,9], and other methods. The detection results of this type of method are intuitive and suitable for motors with obvious fault characteristics, but the performance of early mild fault diagnosis in general. The intelligent diagnosis method uses the trained model to predict motor failure. They include backpropagation neural networks [10,11], support vector machines [12,13], etc. This method has low accuracy and requires a large number of samples to train the model.

Deep learning is an emerging development field of recent years, which commonly includes the convolutional neural network (CNN), stack self-coding machine (SAE), recurrent neural network (RNN), and deep belief network (DBN) [14,15,16,17]. Deep learning has been applied in the field of motor fault diagnosis by its powerful nonlinear mapping ability. Jiang used multiscale unsupervised learning directly from the original vibration signal in learning useful features, to obtain rich and complementary failure mode information on different scales [18]. Kerboua Adlen, through the length of the two memories superimposed, formed a single layer of end-to-end network, trained in the original time series signal complex time relationship. The experimental results show that it has good robustness and real-time. The experimental results show that under different speeds and loads, the method can accurately detect fault types, which is feasible and effective [19]. Zhuang input four features with high classification rate into the RNN network. The experimental results show that the method can accurately detect the type of failure under different speeds and loads, which is feasible and effective. [20]. Ince used a shallow layer and adaptive one-dimensional CNN for real-time detection and classification of damaged rotor rows in induction motors [21]. Shu obtained the vibration signal of the motor through a wireless sensor, converted the obtained vibration signal into an image signal by wavelet transform, and then, used the histogram to equalize and enhance the processed image as the input of CNN. The test results show that the neural network method and several traditional methods have higher accuracy and real-time [22]. Wang preprocessed the original signal with a short-time Fourier-transform to obtain the corresponding time-frequency diagram. Then, CNN adaptive time-frequency feature extraction is used to diagnose the motor fault [23]. Jian constructed a cascading automatic encoder network to extract fault features of input data and improved the fault identification capability of the network by introducing random noise [24]. Currently, the main object of motor detection is ordinary motors, while the detection of micro-special motors is rare.

In this paper, by combining the improved LSTM neural network with the reconstructed signal of wavelet packet, the detail quantity is obtained by subtracting the reconstructed low-frequency signal of the third layer of wavelet packet from the original signal. The detail quantity is used as the character input of the improved LSTM neural network. Experimental results verify the feasibility of the method for eight fault classifications of vibrating motors.

## 2. Motor Working Principle and Fault

### 2.1. Working Principle of Vibration Motor

**Figure 2:**
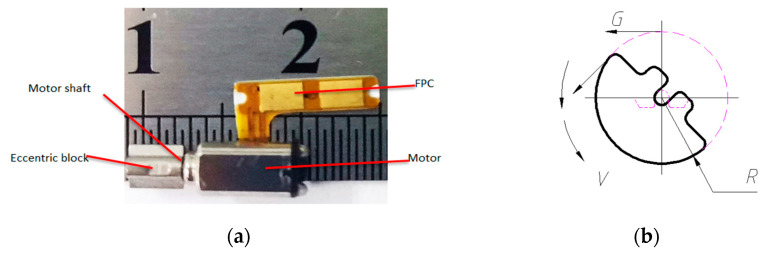
(**a**) Eccentric vibrating motor. (**b**) Eccentric block of eccentric vibrating motor.

The working principle of the eccentric vibration motor is that the power source and the vibration source of the motor are combined to form an excitation source. The shaft end of the vibration motor is equipped with an eccentric block. The center of gravity of the eccentric block and the motor axis is not on the same axis. After being energized, the motor is in an unstable state, and the rotation of the motor shaft drives the eccentric block to generate an inertial excitation force, which is a space rotation force [25], as shown in Figure 2.

At present, the vibration amount of the motor in industry is measured by calculating the acceleration of gravity of the motor. The G value is calculated as follows:

(1) $$G=\mathrm{me}\omega^{2}$$

m,e,and ω  respectively represent the mass of the eccentric block, the distance of the center of the mass rotation axis of the eccentric block, and the frequency of the motor rotation angle. It can be seen from the formula that the polarization force of the motor is proportional to the square of the angular velocity. The greater the velocity is, the greater the polarization force becomes. 

### 2.2. Fault is Introduced

During the processing, manufacturing, and assembly of vibrating motor parts, the motor will produce eight kinds of common faults, such as armature sticking, phase disconnecting, brush faults, wave falls, wave heights, wave lengths, magnetic field faults, and armature confusion. Among them, three kinds of defects, such as armature sticking, phase disconnecting, and armature confusion are fatal and must be 100% detected strictly in the production process. The specific waveform generation reasons and corresponding waveform failure diagrams are shown below.

**Figure 3:**
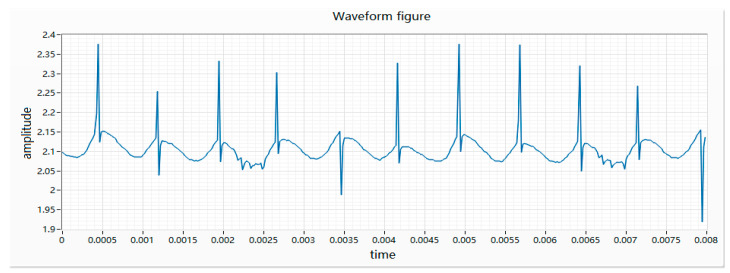
Waveform of a good product.

**Figure 4:**
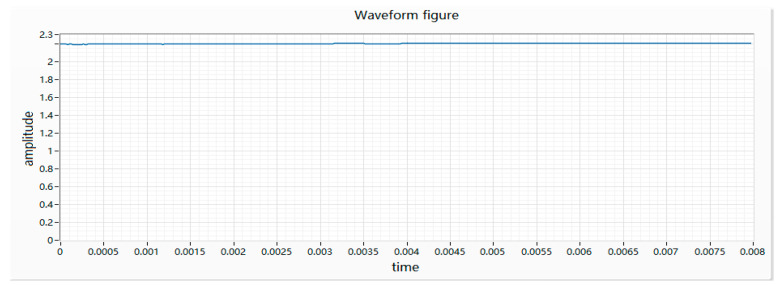
Waveform of a motor armature sticking.

**Figure 5:**
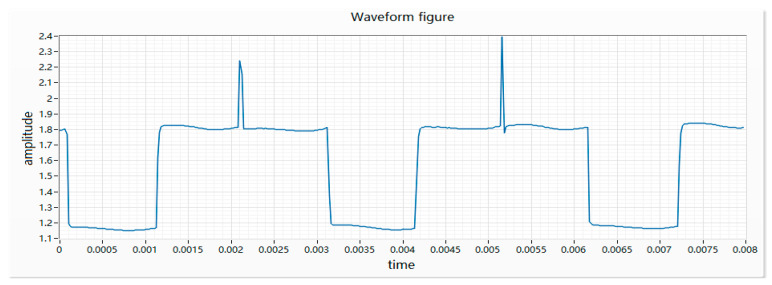
Waveform of phase disconnecting.

**Figure 6:**
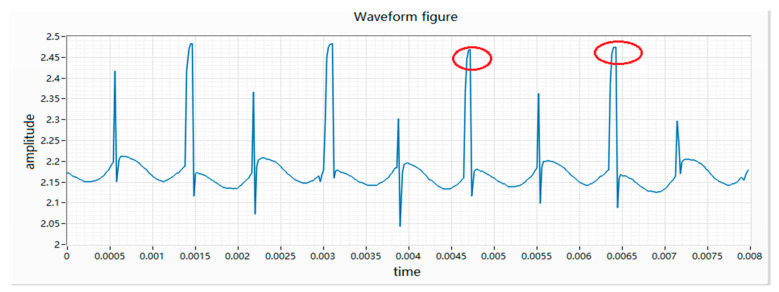
Waveform of a defective brush.

**Figure 7:**
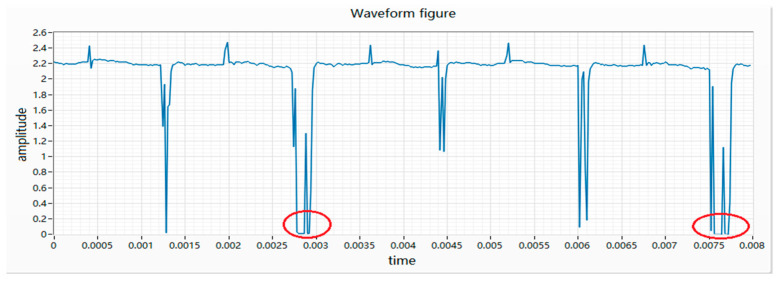
Waveform diagram of waveform fall.

**Figure 8:**
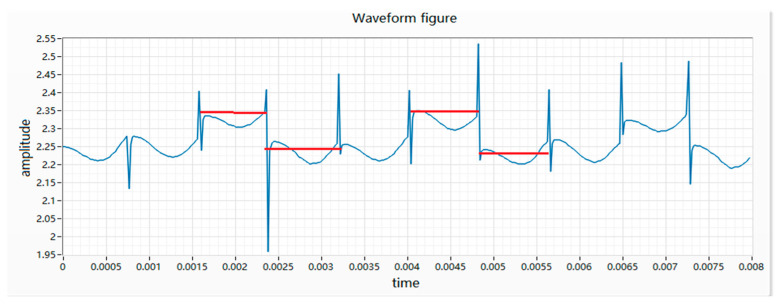
Waveform diagram of wave height.

**Figure 9:**
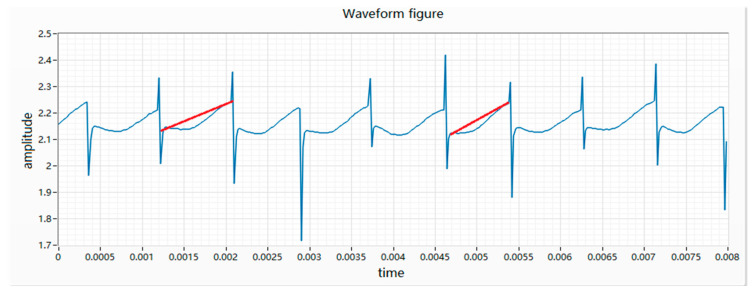
Waveform diagram of a magnetic field fault.

**Figure 10:**
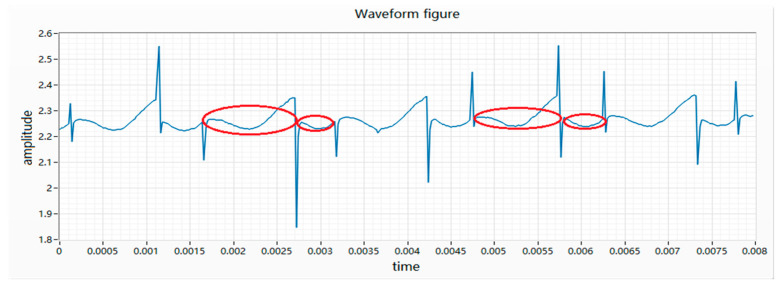
Waveform diagram of waveform length.

(1) During the rotation of the motor, the brush and the pole piece of the motor will be “opened” once every 60 degrees of rotation. At the instant of “opening”, the loop resistance becomes smaller, resulting in an instantaneous current increase. There will be a peak every 60 degrees. Due to the insufficient circularity of the commutator and the commutator process not meeting the requirements, some peaks will be downward. The waveform of the good product is shown in Figure 3.

(2) The armature is stuck as the motor does not rotate after receiving power, and appears as a straight line with a small floating on the waveform. There are various reasons for the sticking, such as the bending of the main shaft, the interference of the bearing, the interference of the rotor and the casing, etc., which causes the motor to be connected to the circuit as a fixed-value resistor. Therefore, the collected waveform fluctuation is small, as shown in Figure 4. 

(3) Phase disconnecting is when a phase of the winding machine breaks a phase during winding or one phase is disconnected during assembly. When the motor is turned to this phase, the entire circuit is equivalent to accessing a fixed-value resistor, as shown in Figure 5.

(4) The failure of the motor brush is due to the bending of the brush or the quality of the motor brush itself during the assembly process. The time during which the brush contacts the armature will become longer, resulting in a split-off phenomenon on the waveform at the moment of commutation, as shown in Figure 6.

(5) The characteristic of the wave fall is that at a certain moment of rotation, the motor is momentarily disconnected, causing the voltage value across the resistor to be collected to be 0. The essence of the waveform drop is the moment when the commutator does not contact the electrode during the movement, which causes the circuit to open. Therefore, the voltage value collected by the acquisition card is 0, as shown in Figure 7.

(6) The difference in height is characterized by the difference in height between two adjacent peaks and the voltage difference is greater than 0.12 V. The essence of the difference in height is that when the rotor of the vibration motor is wound, the winding resistance of two adjacent coils is different, resulting in different resistance values for each phase. After the power is turned on, the motor will turn to this place, which will cause the resistance of the whole circuit to change and the waveform will appear high or low, as shown in Figure 8. 

(7) Magnetic field fault is caused by the fact that the magnet cannot reach the saturation state of magnetization when it is magnetized. After the motor is energized, the internal magnetic field distribution is abnormal, causing the waveform to skew to the left or right, as shown in Figure 9.

(8) The abnormality of the waveform is characterized by the difference in length between the two adjacent peaks. When the ratio of the length of the long section to the length of the short section is greater than 1.3, it is a defective product. The essence of the waveform abnormality is that the two adjacent phases have inconsistent commutation times. In the process of rotation, there is one phase damping is which too large, resulting in a long-time side of the commutation. The waveform collected by the acquisition card is long and short, as shown in Figure 10.

(9) The same production line may adjust the production of other types of motors at any time so that there will be a risk of confusion in the motor rotors with different resistance values, and different rated motors have different rated voltages. Therefore, if a small resistance value is mixed in, the vibration of the motor mounted on the mobile phone will be too large, which will cause discomfort to the human body. If a large resistance rotor is mixed in the motor, it will cause the human body to feel that the amount of vibration is relatively small, and the human body cannot feel the prompt of information. Therefore, the resistance of the motor must be strictly controlled within a certain range. The resistance of the motor studied in this article is 28 ± 2 ohms.

## 3. Related Works and Foundations

### 3.1. Wavelet Packet Theory

The collected voltage signals are non-steady-state signals, including the transient and fault transients of the eccentric rotation of the motor. It is not only difficult to directly identify the fault transients from the measured original signals, but also the identification effect is general. Therefore, using wavelet packet decomposition is an effective method to extract fault transients. The wavelet packet transform decomposes the signal on multiple scales. The wavelet packet can “adaptively change” the structure of the time-frequency window. The appropriate high-frequency and low-frequency parts of the signal are selected for analysis. The variable time window makes the time-frequency window narrower in the low-frequency part and wider in the high-frequency part. The wavelet transform solves the lack of Fourier-transform in the time dimension [26].

**Figure 11:**
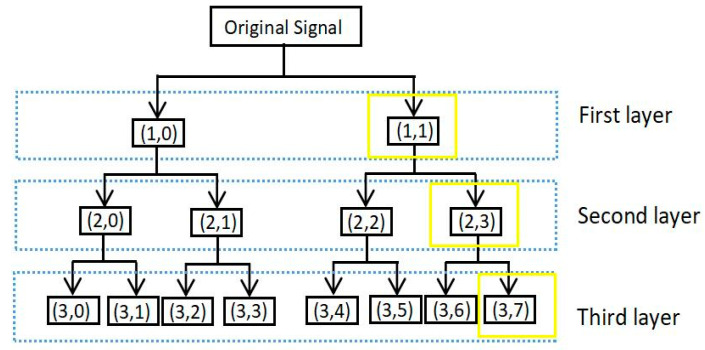
Flow chart of wavelet packet decomposition and reconstruction.

Wavelet packet multi-scale analysis decomposes the entire space into orthogonal sums of multiple subspaces according to different scale factors  j(1,2,3⋯n), as shown in Figure 11. Unified orthogonal decomposition of scale subspace $V_{j}$ and wavelet subspace $\;W_{j}$ into $V_{j+1}$ is [27]:

(2) $$V_{j+1}=V_{j}⊕W_{j}$$

$V_{j+1}\;$ can be shown in a single table $U_{j}^{n}$, and $U_{j}^{n}\;$ is defined as a function. 

$U_{2n}\left(t\right)$, then, the whole space satisfies the double-scale equation

(3) $$\{\begin{array}{c} U_{2n}=\sqrt{2}\sum_{k\in z}h_{k}u_{n}\left(2t-k\right)\\ U_{2n+1}=\sqrt{2}\sum_{k\in z}g_{k}u_{n}\left(2t-k\right) \end{array}$$

In the formula, $h_{k}\;$ is the high-pass filter bank of wavelet packet and $g_{k}\;$ is the low-pass filter bank of the wavelet packet.

During fault feature selection and feature extraction, the intra-class dispersion should be as small as possible, and the inter-class dispersion should be as large as possible. The current signal generated by the motor’s rotating motion is similar to the periodic signal. The original signal is subtracted from the envelope to obtain the reconstruction. According to the principle of permutation entropy, the smaller the entropy value is, the more ordered the signal, the larger the entropy value, and the more disordered the signal is. Since the fault current signal is generated due to the periodic friction between the brush and the pole piece, the smaller the entropy value, the more it can reflect the fault information of the motor [28]. Several common wavelet base entropy values are selected for comparison.

According to the comparison of data in Table 1, the Shannon entropy value after bior2.2 wavelet decomposition and reconstruction is the smallest, it is more orderly, and the details after decomposition contain the most feature information.

### 3.2. Improved LSTM Network

**Figure 12:**
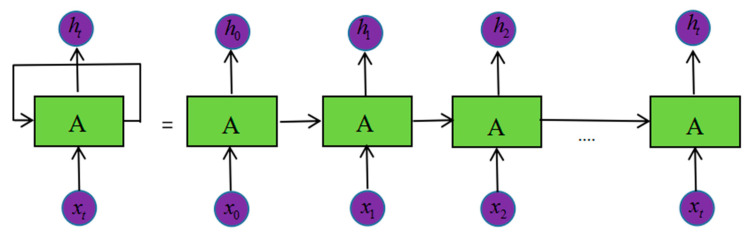
RNN network structure.

The recurrent neural network is mainly used in time series prediction and natural language processing. It is a neural network that automatically models sequence data. One of the key points of RNN is that it can be used to connect previous information to the current task, Thus, the output of the current sequence is related to the input of the previous sequence. As shown in Figure 12, from the network structure, the recurrent neural network will remember the previous information and use the previous input information to affect the output information of the following nodes. However, for a long sequence of networks, the RNN loses the ability to learn to connect to information far away as the spacing increases [29,30].

**Figure 13:**
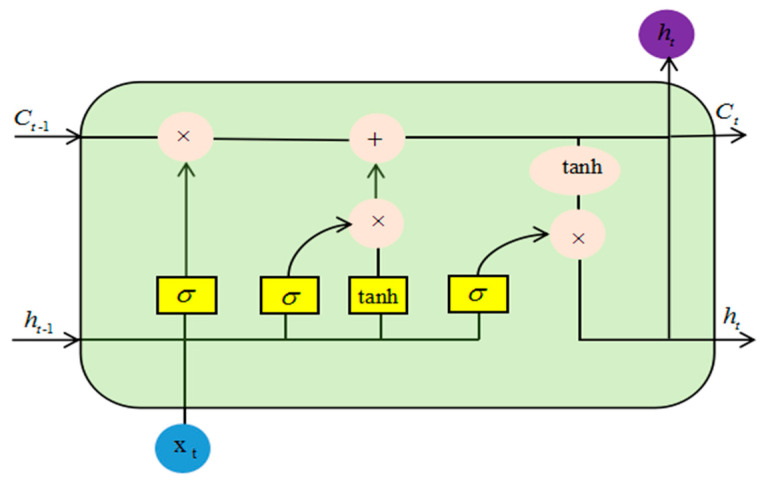
LSTM network structure.

The LSTM neural network is a variant of the RNN network, which was first proposed by Hochreiter [31]. The improved LSTM network based on RNN can well solve the problem of gradient explosion and gradient disappearance. There are one or more cells in each LSTM neuron to record the current state information of LSTM neurons. Besides, there are three control gates in the LSTM network: the Forget Gate, the Output Gate, and the Input Gate, as shown in Figure 13.

The forget gate in LSTM can calculate the information that needs to be forgotten by calculating the selectivity. By using the Sigmoid function, a probability value between 0 and 1 is obtained; 1 represents all reservations and 0 represents all forget.

(4) $$f_{t}=\sigma \left(W_{f}\cdot \left[h_{t-1},x_{t}\right]+b_{f}\right)$$

The formula  σ  represents the Sigmoid activation function, $w_{f}$ represents the weight matrix corresponding to the forgetting gate, $h_{t-1}$ represents the output of the previously hidden layer unit, $x_{t}\;$ is the input of the current moment, and $b_{f}\;$ is the bias term of the forgetting gate.

After deciding which bits of information to discard, the next step is to determine what new information needed will be stored in the cell state. There are two parts to this. The first part of the Sigmoid layer called the “input gate layer” determines what values will be updated. The second part of the tanh layer creates a new candidate value vector that will be added to the state. After deciding which bits of information to discard, the next step is to determine what new information will be stored in the cell state.

(5) $$i_{t}=\sigma \left(W_{i}\cdot \left[h_{t-1},x_{t}\right]+b_{i}\right)$$

(6) $$\tilde{C_{t}}=\tan h\left(W_{c}\cdot \left[h_{t-1},x_{t}\right]+b_{c}\right)$$

$i_{t}$ represents how much input to the current time needs to be stored into the cell state of the current time, $b_{i},b_{c}\;$ is the corresponding offset, and $\tilde{C_{t}}\;$ represents that adding the current time input generates new information into the cell state.

The forget gate and the memory gate determine the updated information, and then, the old cell state can be updated accordingly. The Sigmoid layer determines which part of the cell state will be output. Finally, the cell state is processed by tanh to obtain a value between −1 and 1 to determine the final output.

(7) $$C_{t}=f_{t}\times C_{t-1}+i_{t}\times \tilde{C_{t}}$$

(8) $$O_{t}=\sigma \left(W_{o}\left[h_{t-1,}x_{t}\right]+b_{o}\right)$$

(9) $$h_{t}=O_{t}\cdot \tanh\left(C_{t}\right)$$

In the formula, $C_{t}$ is the hidden layer state, $W_{O}$ is the weight matrix of the output gate unit corresponding to the input $h_{t-1}$ and $\;x_{t}$, $b_{o}$ is the bias term, and $O_{t}\;$ is the output value of the output gate unit.

**Figure 14:**
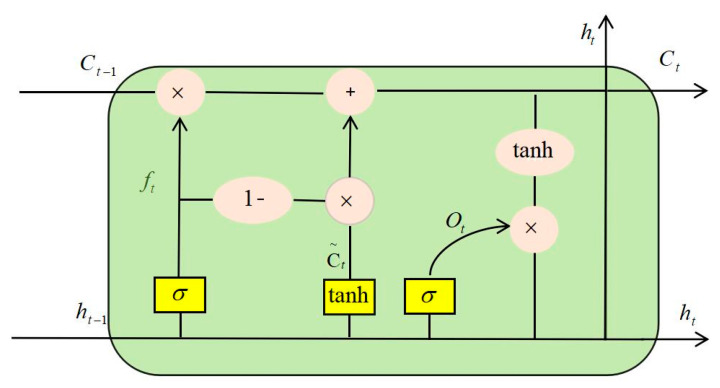
Improved LSTM network structure.

In recent years, LSTM has achieved good results in signal fault diagnosis, but LSTM still has great shortcomings. For example, many parameters can be adjusted, and it is difficult for LSTM networks to converge. Many people have improved LSTM based on their data models [32]. This article proposes an improved structure based on LSTM. The specific structure is shown in the following Figure 14:

(10) $$C_{t}=f_{t}*C_{t-1}+\left(1-f_{t}\right)*\tilde{C_{t}}$$

The input gate was canceled and the amount of new information was added. The amount of old state reserved was set to two complementary values of 1. Thus, we only forget when we needed to add new information; we added new information only when we needed to forget it.

In the classification task, if a sample is far from the number of samples in other categories, the classifier in this case usually performs poorly. In the actual production of the factory, the total number of defective products accounts for less than 2% of the total production, which has led to an extremely uneven distribution of the data of good products and defective products. The loss of the traditional two-class crossover is shown in Equation (11); $y^{,}\;$ is the output of the activation function, so the value is between 0 and 1, and y is the label value. It can be seen that ordinary cross-entropy calculations have a higher output probability for positive samples. The smaller the loss value becomes, and the smaller the output probability for negative samples, the smaller the loss is. This causes the loss function to be slow during the iteration of a large number of simple samples and may not be optimized the best.

(11) $$L=\{\begin{array}{c} -\log\;y^{,}\;\;\;\;\;\;\;\;\;\;\;\;\;\;\;\;\;\;\;\;y=1\\ -\;\log\left(1-y^{,}\right)\;\;\;\;\;\;\;\;\;y=0 \end{array}$$

This paper proposes a loss function that can automatically adjust the risk penalty factor. This method can increase the mining of difficult-to-classify samples and can also adjust the weighting factor to reduce the unsatisfactory classification effect caused by sample imbalance, to improve the accuracy of fault diagnosis.

(12) $$L_{fl=}\{\begin{array}{c} -\alpha {\left(1-y^{,}\right)}^{\gamma }\log\;y^{,}\;\;\;\;\;\;\;\;\;\;\;\;\;\;\;\;\;\;\;\;\;y=1\\ -\left(1-\alpha \right)y^{,}{}^{\gamma }\log\;(1-y^{,})\;\;\;\;\;\;\;\;\;\;y=0 \end{array}$$

α  is a balance factor, which is used to balance the uneven proportion of positive and negative samples. γ  adjusts the loss of easy-to-classify samples so that more attention is put upon difficult and misclassified samples during training. The larger the weight is, the better the accuracy is; or otherwise, the sample with a small probability of occurrence will be more misjudged.

### 3.3. Data Collection and Processing

**Figure 15:**
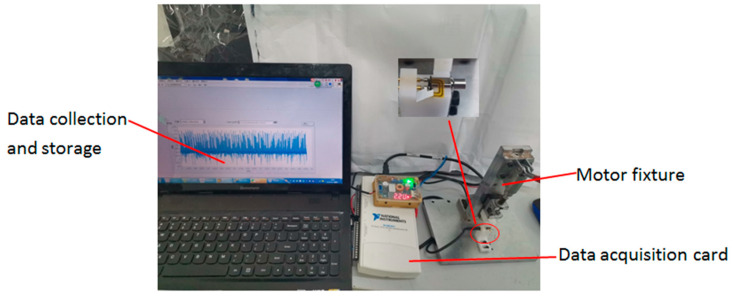
Data acquisition chart.

The acquisition card was more convenient for collecting voltage signals, therefore, it was necessary to add a sampling resistor in the acquisition circuit to convert the current signal into a voltage signal. The resistance value can neither be too large nor too small. Excessive resistance will affect the motor power. The heating of the resistor will also increase during work. If a small sampling resistor will reduce the output voltage of the resistor, the proportion of the collected signal error offset and interference noise will increase. It thereby reduces the sampling accuracy. After many tests and comparisons, a resistance of 30 ohms was selected to obtain better results. The specific collection device is shown in Figure 15.

The data acquisition of the experiment used LabVIEW2018 software, the acquisition card used NIUSB-6211, and the sampling rate was 50 K/s. We collected the data of nine types of motor: armature sticking, phase disconnecting, brush fault, wave fall, wave height, wave length, magnetic field fault, armature confusion, and good quality motor, including 500 good motors. Bad samples collected 100 motors each, the acquisition time was 0.48 s, and the number of sampling points was 24,000. The rotation frequency of each motor was around 220 Hz. Then, the number of sampling points per rotation cycle:

(13) $$N=\frac{1}{f}*f_{s}$$

In the above formula, N is the number of points collected when the motor rotates for one revolution, f   is the rotation frequency of the motor, and $f_{s}\;$ is the sampling rate set by the acquisition card.

Selecting 240 points generally includes one complete waveform cycle. Due to the non-stationary of the motor during the rotation, even if the same motor is rotating at different rotation moments, the waveforms collected will be slightly different. Therefore, the same motor can be used for continuous sampling, and then, the data obtained can be segmented. The data were entered as a sequence every 240 points. The data format was (6, 40). The collected data were divided according to 240 points. The total number of good products in the dataset was 50,000 samples, and the total number of defective products in each category was 10,000. We used a 4:1 ratio of the training set and test set, as shown in Table 2.

## 4. Results

**Figure 16:**
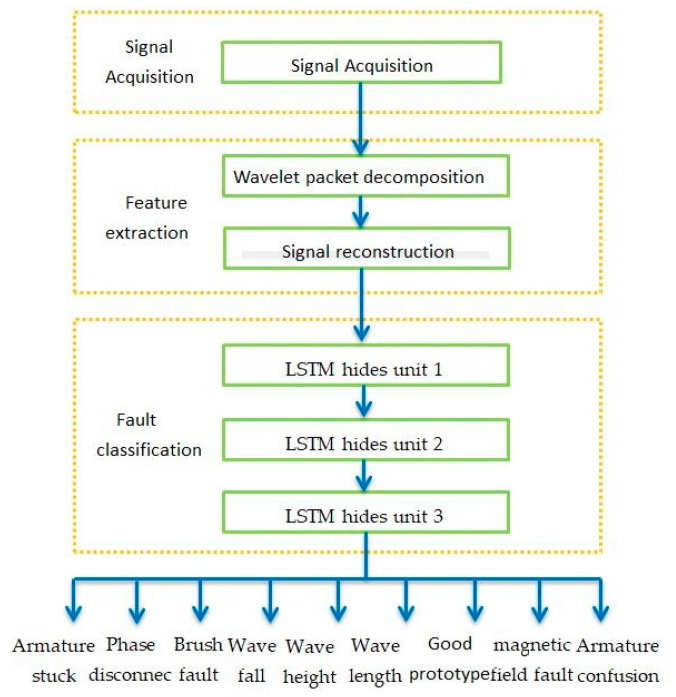
Algorithm flow chart.

The test and training were done on a Win10 system, the hardware used a GTX2080ti graphics card, the software language was Python 3.7.0, the compilation environment was Pycharm 5.0.3, and TensorFlow version was 1.9.0. The program flowchart is shown in Figure 16.

It is difficult for a single-layer improved LSTM network to learn the time-series characteristics of high-dimensional data, while a multilayer LSTM network uses the network output value of the upper layer as the input value of the next layer. It makes the learning ability of the multilayer improved LSTM network stronger. However, as the number of layers increases, the network training time increases, which will increase the difficulty of data convergence. Therefore, the number of layers of the network has an important impact on the accuracy of the fault diagnosis. With the remaining parameters unchanged, only the LSTM network layer number was changed for a control experiment. The experimental results are shown in Table 3.

The number of wavelet packet decomposition layers is another important parameter for using the wavelet packet decomposition algorithm. The wavelet packet decomposes the original signal step by step. The i-th level will have a $2^{i}$ power node and each node corresponds to a wavelet packet coefficient. This decomposition coefficient determines the distribution of wavelet energy and frequency band. If the number of layers to be decomposed is too small, the energy and frequency band will be very concentrated, and it is impossible to determine the frequency band in which the fault signal is located accurately. The decomposition of sub-signals into different frequency bands will also increase the amount of calculation. Therefore, in actual engineering practice, it is necessary to consider the frequency domain resolution and time domain resolution to determine the number of wavelet packet decomposition layers [33]. Here, we separately used the original data and the wavelet packet to decompose the reconstructed signal after 1–4 layers as the original input of the LSTM network. As shown in Table 4, through comparison, it was found that the accuracy of the signal reconstruction after wavelet packet decomposition of three layers was higher. 

By comparing the three algorithms of LSTM, improved LSTM, and SVM, by continuously adjusting the parameters, we found that the improved LSTM still showed the highest accuracy rate, as shown in Table 5.

**Figure 17:**
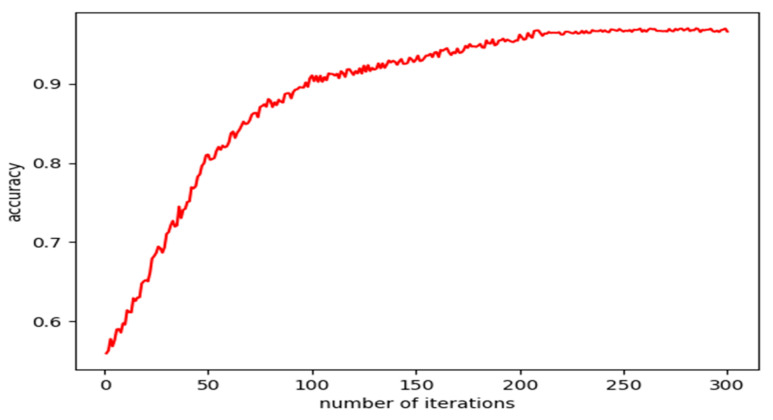
Relationship between the number of iterations and accuracy.

An important parameter that affects the accuracy of LSTM network training is the number of iterations. With the remaining parameters unchanged, only the number of iterations was changed and a control experiment was performed. The experimental results are shown in Figure 17.

It can be seen from Figure 17 that the number of iterations had a positive correlation with the accuracy of the LSTM network, but the accuracy did not change after the number of iterations reached about 300. Therefore, the number of iterations of the network was set to 300. The rest of the hyper-parameters, parameters such as batch size and learning rate, are shown in Table 6.

We used the LSTM network parameters in Table 6 to train a three-layer improved LSTM network model on the TensorFlow framework and used the trained model to perform real-time testing. The overall accuracy rate of the testing set was 96.58%. The predicted value was further compared with the true value of the test set. The results are shown in Table 7. 

In the final statistics, there were 40 defective products mixed in the good products, the accuracy of which was 99.6%, and no fatal defects were included. We used the manual measuring device shown in Figure 15 to measure more than 40,000 vibration motors, and the results show that the accuracy of the system is reliable. At the same time, the accuracy and speed of detection are higher than the previous measurement methods.

## 5. Conclusions

Based on the improved WP-LSTM, this paper proposes an efficient and accurate fault diagnosis method for miniature vibration motors. This method decomposes the three-layer wavelet packet of the motor voltage signal data and reconstructs the signal. The reconstructed signal is input as a feature vector into the three-layer improved LSTM network, and the improved LSTM network is used to learn the signal. Finally, the trained network model is used to diagnose the motor in real-time. The effectiveness of the inspection method increases. This automatic inspection equipment can not only achieve good quality control for the production of the factory, but also saves labor costs and produces practical benefits for the factory. At the same time, the automatic testing equipment fills the limitation of the vibration motor relying on manual testing and promotes the development of miniature vibration motor testing.

## 6. Patents

The results of the associated with this article, three invention patents and software copyright, respectively, are the miniature vibration motor defects based on convolution neural network fault classification method and the device, authorized number: CN201910263769.5, a miniature type vibration motor current fault diagnosis instrument and diagnosis methods, authorized number: CN201910382783.7, software copyright: miniature motor fault classification V1.0, authorized number: 2019SR0158885.

## Figures and Tables

**Table 1:** There are five common entropy values of wavelet-based instances

Wavelet Base	haar	db3	Bior2.2	Coif2	Symlets
Entropy	3.01	2.84	2.24	2.76	2.57

**Table 2:** Dataset.

Parameter	Good Samples	Bad Samples
Total dataset sample	50,000	80,000
Training set samples	40,000	64,000
Test set sample	10,000	16,000

**Table 3:** LSTM network layer comparison results.

LSTM Network Layer	Accuracy/(%)
1	80.24
2	90.46
3	96.58
4	95.64

**Table 4:** Comparison results of wavelet packet decomposition layers.

Wavelet Packet Decomposition Layers	Accuracy/(%)
0	74.20
1	92.24
2	95.71
3	96.58
4	96.32

**Table 5:** Comparison of accuracy between different algorithms.

Algorithm Model	Accuracy/(%)
SVM+WP	80.72
LSTM	89.35
LSTM+WP	94.98
improved LSTM+WP	96.58

**Table 6:** LSTM network parameters.

Parameter	Numerical Value
LSTM network layer	3
LSTM hidden layer 1	256
LSTM hidden layer 2	128
LSTM hidden layer 3	9
Learning rate	0.001
Iterations	300
Batch size	256
Node	(3,7)

**Table 7:** Confusion Matrix.

Confusion Matrix	Predicted Positive	Predicted Negative
True Positive	9535	465
True Negative	393	15607
